# Correction of gas chromatography–mass spectrometry long-term instrumental drift using quality control samples over 155 days

**DOI:** 10.1038/s41598-025-24794-y

**Published:** 2025-11-19

**Authors:** Jie Yu, Tong An, Daifeng Chen, Shining Zong, Dongxiao Bai, Dawei Qi, Luning Zhang, Junming Shi

**Affiliations:** 1 Technical Center, Shanghai Tobacco Group Co. Ltd., Shanghai, 201315 China; 2 School of Chemical Science and Engineering, Tongji University, Shanghai, 200082 China; 3 Shanghai Cigarette Factory, Shanghai Tobacco Group Co. Ltd., Shanghai, 201315 China

**Keywords:** Biological techniques, Chemistry

## Abstract

Long-term instrumental data drift is a critical challenge for ensuring process reliability and product stability. In this study, we conducted 20 repeated tests on the smoke of six commercial tobacco products using gas chromatography-mass spectrometry (GC–MS) instrument over 155 days. We propose a simple, cost-effective, and reliable peak-area correction approach to address long-term data drift, especially on GC–MS data. Using 20 pooled quality control (QC) samples, we establish correction algorithm data set, and achieved reliable peak correction even for compositions exhibiting large fluctuations. Three algorithms − spline interpolation (SC), support vector regression (SVR), and Random Forest (RF) − were applied to normalise 178 target chemicals in 20 repeated measurements on six samples. Two key innovative approaches were introduced. First, we established a “virtual QC sample” by incorporating chromatographic peaks from all 20 QC results, via retention time and mass spectrum verification. This virtual QC served as the meta reference for analyzing and normalizing the test sample. Second, we took translated batch effects and injection order effects into two numerical indices in the algorithms, minimizing artificial parameterization of experiments. For chemical components found in the test samples but absent in the QC samples, normalisation was achieved using either adjacent chromatography peak for correction or by applying average correction coefficients derived from all QC data. Our results show that Random Forest algorithm provided the most stable and reliable correction model for long-term, highly variable data. Principal component analysis (PCA) and standard deviation analysis confirmed the robustness of the correction procedure. In contrast, models based on SC and SVR algorithms exhibited less stability with SC being the lowest. For data with large variation, SVR tends to over-fit and over-correct. This study shows that for long-term data measurements by GC–MS, periodic QC sample measurements combined with appropriate algorithm for correction can effectively compensate for long-term measurement variability, enabling reliable data tracking and quantitative comparison over extended periods.

**Supplementary Information:**

The online version contains supplementary material available at 10.1038/s41598-025-24794-y.

## Introduction

In many applications, samples analysis can require long cycles to complete. In recent years, automated synthesis robots and automated detection robots have rapidly advanced, largely supported by artificial intelligence (AI)^1–3^. These advances are primarily driven by the rapid development and automation of instrumental analysis technologies. For example, in 2020, Burger and colleagues used a robot in conjunction with automated gas chromatography, which ran continuously for 8 days to successfully complete 688 experiments. The robot efficiently prepared high-quality photocatalysts^4^ using a grouped Bayesian search algorithm for experimental parameters selection. Similarly, in 2023, Szymanski and coworkers developed a robot with combined machine learning and automated X-ray powder diffraction analysis. After running continuously for 17 days, they obtained 41 new solid inorganic materials^5^.

In extended quantitative analyses, long-term quality control of signal is a critical factor to ensure repeatability and reliable decision-making. Chromatography-mass spectrometry method is widely used in both research laboratories and industrial settings. However, detecting long-term data drift and performing appropriate correction is particularly important yet difficult for such measurements^6^. During routine operations, many factors can alter or attenuate chromatography and mass spectrometry signals, including instrument power cycling, column replacement, mass spectrometer tuning, ion source cleaning, filament replacement, and quadrupole cleaning^7,8^.

Currently, attention on normalisation of chromatography and chromatography-mass spectrometry data mostly focuses on metabolomics samples^7–17^. The published methods can broadly be classified into three types. The first one relies on quality control (QC) sample normalisation. In principle, QC should contain all chemicals from all samples. A widely used approach is to pool aliquots from all samples to be analysed into a composite standard QC^7,9,15–17^. By measuring the QC at regular intervals over time, a normalisation curve or algorithm can be established for other samples. The second one is based on internal standard (IS) to do sample normalisation, in which regularly measured IS containing a few typical compounds are used to establish correction curves over long-term studies^18^. Quantitative analysis of actual samples is then derived from these IS data. The third type is called data-driven. It relies on the intrinsic robustness of a dataset itself with relatively fixed major constituents for normalisation^10,12,13^.

QC-based correction relies on the assumption that all components (i.e., specific molecules) present in the samples are also found in the QC samples, a condition that requires calibration over time. Thus, QC measurements at different experimental stages provide a basis for long-term calibration. However, the assumption that QC sample stays unchanged with fixed compositions may not be valid^19^. For instance, new compounds may appear in later samples that were not present in the original QC. Or else, after prolonged studies, some components in QC may disappear because the detection limit of instrument had increased. When sample components no longer overlap fully with QC components over extended periods, meaningful correction becomes increasingly important yet difficult.

Several methods have been proposed to address such challenges, particularly in the realm of large-scale untargeted metabolomics. For example, Kuligowski et al. employed support vector regression (SVR) to correct intra-batch effects using QC samples in liquid chromatography-mass spectrometry (LC–MS)^20^. Wang et al. developed a regression calibration method, *Batch Normalizer*, to adjust both batch and injection order effects in LC–MS data^21^. Furthermore, wavelet-based methods, such as those proposed by Deng et al., have shown promise for removing batch effects in untargeted metabolomics data^22^.

More advanced approaches have emerged recently, including the integration of data-driven normalisation with machine learning algorithms. For example, in 2016, Shen et al. demonstrated the successful normalisation of large-scale metabolomics data using support vector regression^23^, while Brunius et al. combined between-batch feature alignment with within-batch signal intensity drift correction to address signal variance across batches^24^. Additionally, a recent study by Wehrens et al. showed that explicitly incorporating batch and injection order information generally yields excellent correction results^25^. Recently, Boccard et al. utilized stratified subsampling of metabolomic data to remove batch effects in endocrine disruptor screening studies^26^.

To address the growing need for accurate and reliable long-term data correction, this study proposes a novel and robust correction protocol based on QC data from GC–MS measurements conducted over a 155-day period. We classify the components in the samples into three distinct categories: (1) Category 1, components present in both the QC and sample; (2) Category 2, components in sample not matched by QC mass spectra, but within the retention time tolerance of a QC component peak; and (3) Category 3, components in sample not matched by QC mass spectra, nor any peak exist within the retention time tolerance window. Three distinct data processing approaches are established for these three cases. We have found that using mathematical data modeling on QC results can achieve reliable quantitative correction for data collected over long time.

In the following, we first summarize theoretical background for data modeling, experimental details, and data processing procedure. Then we present different models used for correction of QC sample results. In the end, optimal data processing and algorithmic model is proposed for long-term signal drift correction.

## Methods

### Data correction theory

The key problem in data correction is translating sample data into quantifiable mathematical parameters. In this paper, two parameters, batch number and injection order number, are used to correlate experimental data with sample properties. Each measurement on QC or a sample is defined with these two parameters. First, the batch number is used to express changes over large time span^11,14,27^. During measurement, switching the instrument on and off can introduce drift, thus switching on the instrument with tuning the mass spectrometer signifies a new batch. Samples measured afterwards before the next instrument turning off all belong to the same batch. The batch number is an integer, designated as “*p*” and starts from 1. During the 155 days for measurements, GC–MS was turned off and on seven times, and therefore batch number is from 1 to 7. The injection order number, an integer designated as “*t*”, is used to identify the sequence of sample measurements within the same batch. Within a batch, all samples were sequentially numbered from 1 to the end of the measurement therein.

We first built our data set using results from pooled QC samples. More detail on experimental process is given in the next section. Suppose one has *n* measurements on QC in chronological order (specifically, 20 measurements in 155 days), and the peak area of component *k* is recorded as {X_i,k_}, *i* = 1,…,n, where *i* represents the sequential order of the QC. First we take the median of the peak areas of component *k* in these *n* measurements as the true value of *k* and denote it as X_T,k_. Then the correction factor for component *k* in the *i*-th measurement of the QC can be written as Eq. 1:

1 $$y_{i,k} = X_{i,k} /X_{T,k}$$

We then express the correction factor y_k_ as a function of the sample batch number *p* and the injection order number *t*, as expressed in Eq. 2:

2 $${\mathbf{y}}_{{\mathbf{k}}} = {\mathbf{f}}_{{\mathbf{k}}} \left( {{\mathbf{p}}, \, {\mathbf{t}}} \right)$$

For component *k* correction, we first calculate a set of correction coefficients {y_i,k_} using the 20 QC data set according to Eq. 1. Subsequently, using {y_i,k_} as the target data set, several algorithms are introduced to find **f**_**k**_**(p, t)** by using batch number {p_i_} and injection order number {t_i_} as the input data sets. In this way, correction function of component *k* is obtained as f_k_. For the correction of component *k* in an actual sample (designated as sample S), one only needs to input the corresponding batch number *p* and injection order number *t* into the function f_k_ to predict its coefficient. When the peak area x_s,k_ (raw data) of component *k* needs correction, we can calculate the corrected peak area x’_s,k_ by Eq. 3:

3 $$x'_{{S,k}} = x_{{S,k}} /y$$

### Data correction algorithms

In order to find **f**_**k**_**(p, t)** using {y_i,k_} as the target data set, three algorithms have been used by using batch number {p_i_} and injection order number {t_i_} of pooled QC samples as the input data sets. The three algorithms are summarized below.

The first one is called Spline Interpolation Correction (denoted as SC). Spline interpolation uses segmented polynomials to deal with the problem of interpolation between data points. In this paper we used the R_bf_ algorithm (scipy.interpolate) in Python’s computing library. The algorithm’s interpolating function offers many choices, but the 3rd and 5th term interpolations fluctuate heavily here, and the linear function is difficult to come up with reasonably good result because of the relatively sparse QC data set. We thus chose Gaussian function.

The second one is called Support Vector Regression (denoted as SVR). Support Vector Regression (SVR) is a variant of Support Vector Machine classification used to solve the problem of numerical prediction of continuous functions. Here the optimal hyperplane for the solution is a regression function, which takes values as close as possible to the data points and makes predictions within certain allowed error.

Python’s machine learning library (sklearn) provides vector regression algorithm. The algorithm offers many adjustable hyperparameters. We here used the leave-one-out cross validation method. We set gamma parameter to be 2^–3^ ~ 2^3^, and chose the exponential increment of 1. We chose gamma value predicted by minimum mean square prediction, and set epsilon parameter after a set of calculations, which gave an optimal value of 0.2. For the regularisation parameter c, we chose a value which is the difference between 90% percentile and 10% percentile.

The third algorithm is called Random Forest (denoted as RF). Random Forest is based on an integrated algorithm consisting of multiple decision trees formed by the resampling technique. It is able to effectively solve random prediction problems^15,28,29^. We used Random Forest algorithm in Python’s machine learning library (sklearn.ensemble). The determinant parameter is n_estimators, which is the number of trees in the random forest. We set the n value to be 100 as suggested by Han et al.^28^.

### Data correction process

The experimental and data correction procedures are shown in Fig. 1. First, we obtained all peak information of a sample (chromatographic retention time, peak area, and mass spectrum), and we verified each constituent using mass spectrum by comparing with that of QC. Upon this comparison, components fell into three categories. In Category 1, the components in the sample can be matched by the components in QC. In this case, correction coefficients can be directly computed by the function developed before. In Category 2, the GC retention time of component *k* is close to the retention time of component *m* in QC within a predefined tolerance window, but none of the mass spectra match. Because GC retention times usually highly correlate with molecular structure and molecular polarity, the correction coefficient predicted by the correction model for component *m* is thus chosen as the correction coefficient for component *k* to be analysed. In Category 3, neither the retention time (within tolerance window) nor the mass spectrum of component *k* in the sample matches anything QC. For such species, the average of correction factors for all components in the QC is used as the correction factor for the component in the sample.

**Fig. 1:**
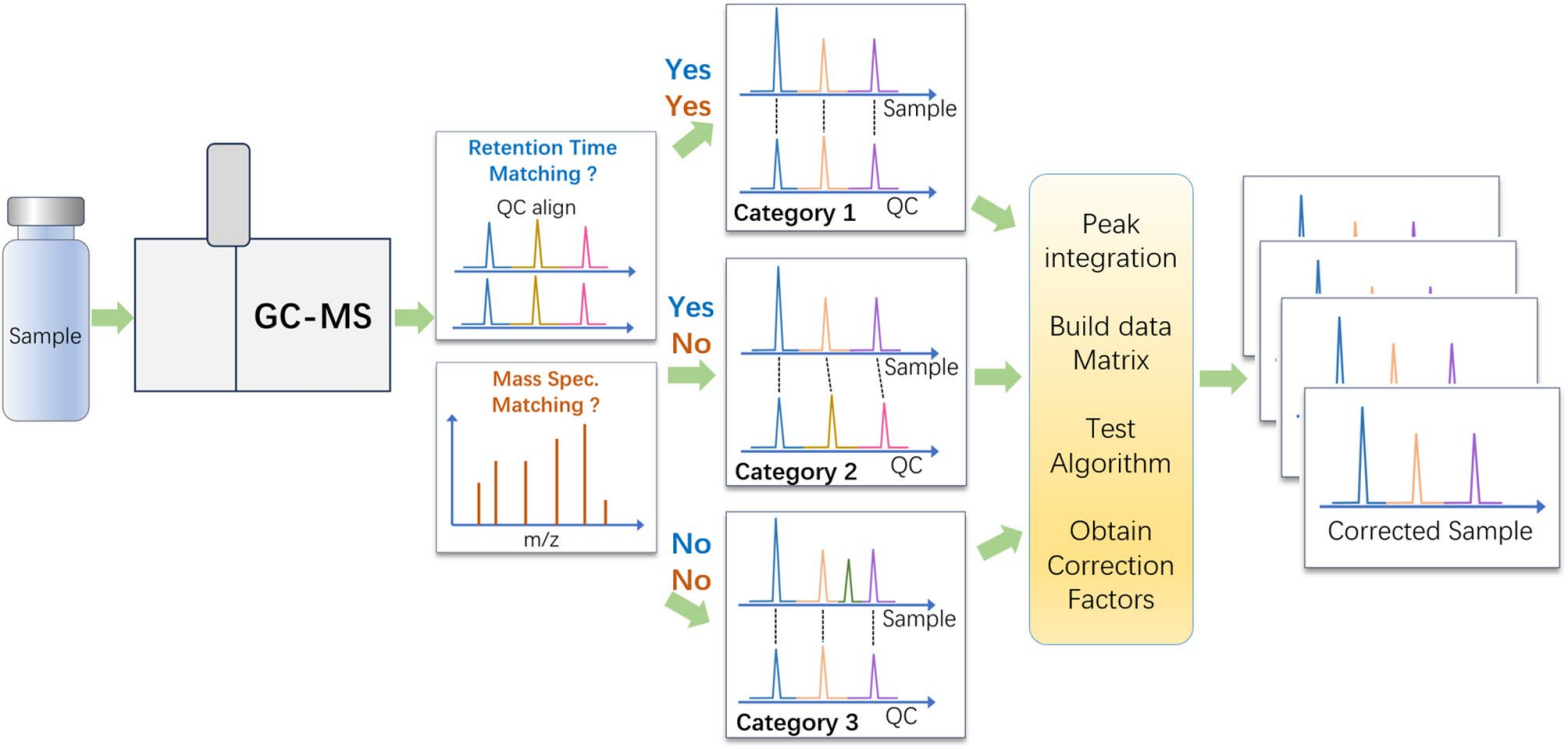
Data measurement and correction procedure. The sample peaks are selected and matched with QC peaks for retention time and mass spectral feature verification. Sample components are classified into three categories based on their retention time and mass spectrum. Different correction process is used for each category.

### GC–MS experiment

Six types of cigarette samples (hereby labeled S1, S2, S3, S4, S5, S6) were smoked on a 20-port smoking machine (Puffman Automatics, Model X200AF, Shanghai, China). The smoking machine was set at 30 mL puff volume with continuous smoking time of 2 s at 60 s interval. Mainstream smoke was trapped on a 92 mm diameter Cambridge fiber pad. This part of the experiment conformed with ISO 3308:2000 and Chinese National Standard GB/T 19,609–2004. The total particulate matters absorbed by the filter pad were extracted with 20 mL of CH_2_Cl_2_ under 30 min of shaking. The CH_2_Cl_2_ solvent was spiked with 100 μL of internal standard (IS) solution (with isobutyl acetate, isobutyl hexanoate, ethyl undecanoate and ethyl heptadecanoate, each at 1.0 mg/mL). In the original experiment design, we wanted to use IS for absolute retention time alignment. However, later on we proposed “tolerance window” approach for peak sequencing. Then mass spectrum comparison was used to identify each unique chemical component. Thus the IS was not used form retention time calibration.

Afterwards, the supernatant of each sample was extracted. Equal volume aliquots of all the six sample solutions were mixed together to form a pooled QC. All QC used in this study was this pooled QC which contained all components from all six samples. A total of 20 vials of each sample were aliquoted and grouped in 20 sets for future injection sequence. Sample preparation and aliquoting were finished at one time on one day, followed by storage at 4 °C for 30 days before analysis. Previous studies indicate that the most significant compositional changes occur in the first week, during which some unstable compounds undergo noticeable degradation. However, the rate of change decreases markedly over time. After one month from sample preparation, the composition generally stabilizes and remains largely unchanged thereafter.

The GC–MS used was Aglient 7890A-5977. The column was two DB-WAX capillary columns (60 m*0.32 μm*0.25 μm) in series. We used Split/Splitless (SSL) injection port with helium as the carrier gas working in constant flow rate mode set at 2.2 mL/min. The sample injection port was set at 280℃ in splitless mode. For programmed heating, the initial temperature was set at 35℃ and kept for 8 min, and then ramped to 230℃ at a rate of 2℃/min, and finally kept for 20 min.

For mass spectrometry, the MS transfer line temperature was at 240℃. Ionization mode was set as EI with 70 eV ionization energy. The ion source temperature was set at 230℃. The quadrupole temperature was at 150℃ with a full scan mode and an m/z range between 33 and 550. A solvent delay time of 20 min was set for solvent elution.

The feed sequence was kept as: blank #1 (solvent only), n-alkane standard, QC, S1, S2, S3, S4, S5, S6, blank #2 (solvent only), totaling 10 runs each time. The same measurement was repeated 20 times over a 155-day span in 7 batches. The chronological timeline of the measurements and the related GC–MS maintenance are shown in Fig. 2. The horizontal axis shows the number of days from the first day. Other tested samples on the same instrument not covered in this paper are omitted for clarity. During the 155 days, all operational actions, such as powering on/off, tuning, replacing filaments, and ion source cleaning, were recorded in detail.

**Fig. 2:**
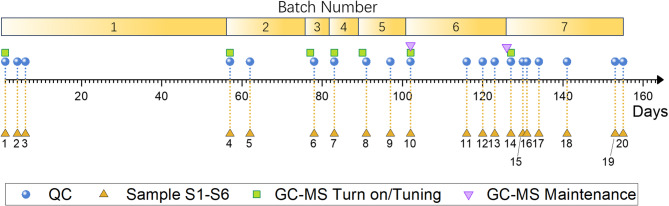
Schematic of chronological QC and sample injection, and instrument operations. The horizontal axis is date of testing, starting on day 1 and ending on day 155. Four types of points represent instrument turn-on and tuning (square), cleaning and filament replacement operation (inverted triangle), measurements on QC (round dots) and samples (triangles, samples S1 to S6). Total 20 sets of measurements are done in 7 batches.

As we mentioned earlier, batch number *p* and injection order number *t* are the two parameters to locate a sample in the data set. Samples are labeled in the following format: sample name—batch number *p*—injection order number *t*. Each GC–MS turn off/turn-on and tuning marked a new batch. All measurements in the same batch were numbered sequentially from 1. If tests other than those needed in this paper were performed, they were also counted in the numbering sequence. This means testing other samples from other projects would change the injection order number *t*, but does not change batch number *p*. This is reasonable since the injection order number is a parameter to define the time lapse effect within a batch. For example, the samples tested on day 155 belonged to batch 7. The QC and S1 were the 185th and 186th samples in this batch, respectively. They were labeled as QC-7–185 and S1-7–186, respectively. All the labels for QC and samples are given in Table S1, with the first column corresponding to chronological time (in days) of the 20 sets of measurements.

### Build virtual QC

Because of signal fluctuation, retention time of components in pooled QC also drifted to various degrees on different dates. In Figure S1 in the supplementary material, we show that QC sample chromatography results vary on different days. In order to establish a reliable retention time standard, a chromatography peak alignment protocol was first established. Our approach is by forming a virtual QC matrix by including all components from all 20 QC runs. Virtual QC is formed as follows.

1. Set the chromatography peak height threshold and retention time error tolerance. We set a GC peak height threshold of 30,000, and judging from retention time drift of some major GC peaks within 155 days, we set the retention time tolerance window to be 36 s. This was typically the largest possible GC drift of a component within 20 QC measurements.
2. Arrange all QC in the order of testing time and take the QC in the middle as the initial virtual QC sample. Determine the retention time and mass spectrum of each chromatographic peak with a height greater than the threshold.
3. Compare the mass spectrum of all chromatography peaks of QC with the initial virtual QC for within the 36 s tolerance range. If the mass spectra comparison is successful, the retention time is taken as the retention time of the current virtual QC. If the mass spectra comparison is unsuccessful, the retention time and mass spectra are recorded and inserted into the current virtual QC sample. When all QCs are analysed, a complete virtual QC sample is thus generated.
4. Compare all QC mass spectra with the final virtual QC, record peak areas, batch numbers, and injection order numbers.
5. Perform similar data manipulation on all samples using the above approach. Align all sample peaks according to the final virtual QC data and check if retention time and mass spectrum match those of QC. Record peak height, batch numbers, and injection order numbers. Label and categorize all sample components in three categories, as mentioned before.

## Results and discussions

### Algorithm verification using QC

After developing models with Spline Interpolation (SC), Support Vector Regression (SVR), and Random Forest (RF) using dataset from 20 QC, we then verified the obtained correction functions on QC data to check algorithm’s performance. The result is shown in Fig. 3. We chose limonene for demonstration, a component appeared in all samples with reasonably strong peak intensity. This molecule appeared at retention time of 36.37 min. In Figure S2, we plot the original 20 chromatograms of QC samples in the 33–50 min elution time. The plot clearly shows the variation of uncorrected data in 155 days.

**Fig. 3:**
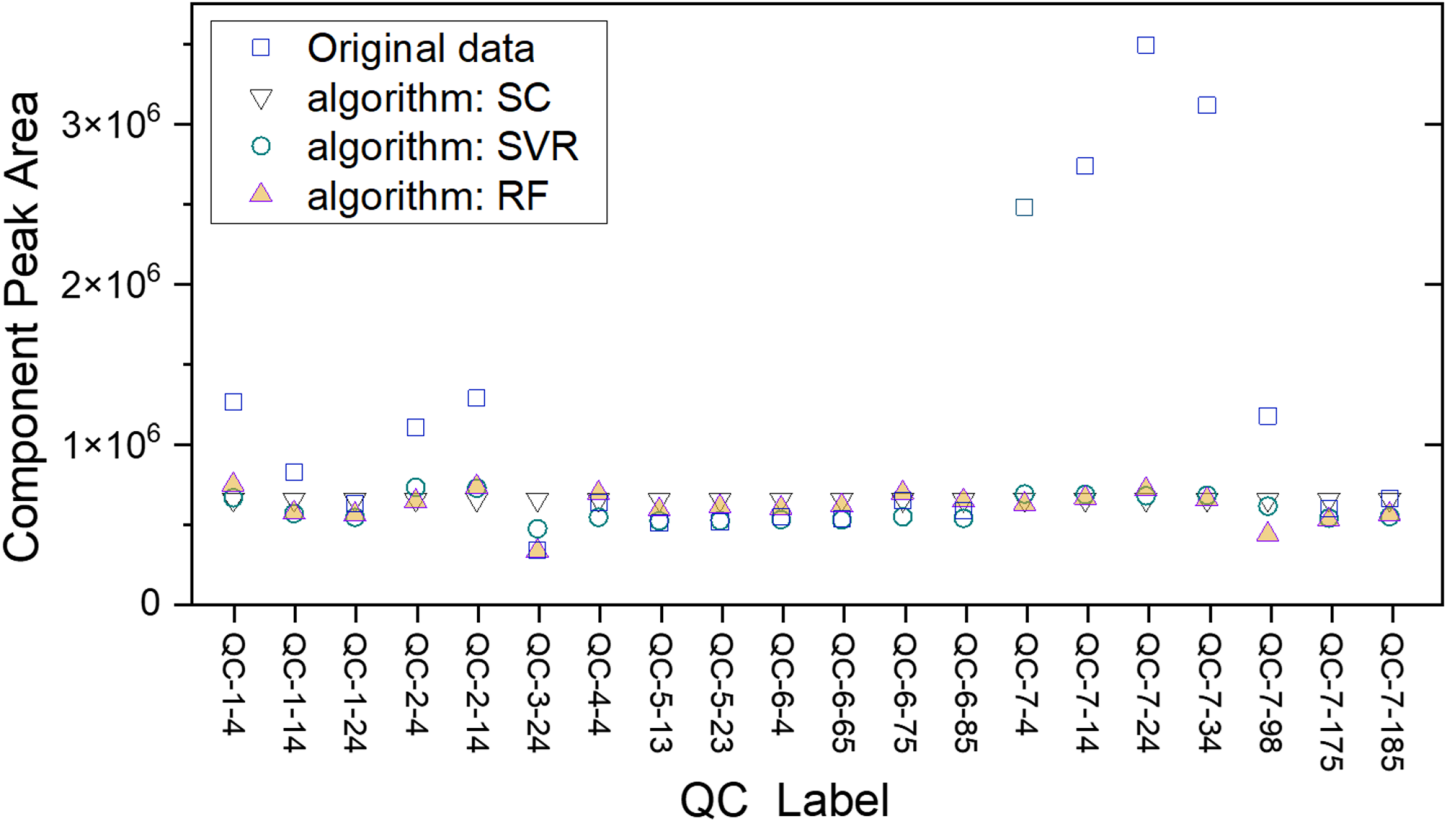
Performance of Spline Interpolation (SC), Support Vector Regression (SVR), and Random Forest (RF) on limonene component in QC. The notations are as follows: square dots are original data, down-pointing triangles are SC, round dots are SVR, and the up-pointing triangles are from RF.

As seen in Fig. 3, the raw data points (open squares) of a few QC varied drastically. All data points became close to one another after correction. Before correction, the peak area standard deviation (SD) of limonene was 9.4 × 10^5^ from 20 tests. The standard deviations after correction using SC, SVR, and RF dropped to 6.8 × 10^4^, 7.9 × 10^4^, and 1.0 × 10^5^, decreasing by 93%, 92%, and 89%, respectively. All three algorithms performed well, in which SVR and SC were slightly better than RF. For QC-7–4, QC-7–14, QC-7–24, and QC-7–34 data points that showed large fluctuations, the correction performed well. Decrease of standard deviation was calculated using the formula: (SD_Original_—SD_Corrected_)/SD_Original_.

### Sample data correction

We then applied the above QC-based correction function on actual samples. We selected limonene of S1 sample for demonstration. In Figure S3, we plot the original 20 chromatograms of S1 samples in the 33–50 min elution time. The plot clearly shows the variation of uncorrected data in the 155 days period. The correction results on limonene peaks are shown in Fig. 4. It is seen that the raw data fluctuated larger than that of QC. After correction, SC algorithm resulted in a some data points that were even worse than the original data, such as the three points of S1-1–25, S1-3–25, and S1-4–5. This suggests that SC is not robust enough for sample correction. Overall, the RF correction results were the most stable. In four measurements with quite large fluctuations (S1-7–5, S1-7–15, S1-7–25, and S1-7–35), the correction by RF was better than both the SVR and the SC results. The good performance of RF agrees with literature results^15,28,29^.

**Fig. 4:**
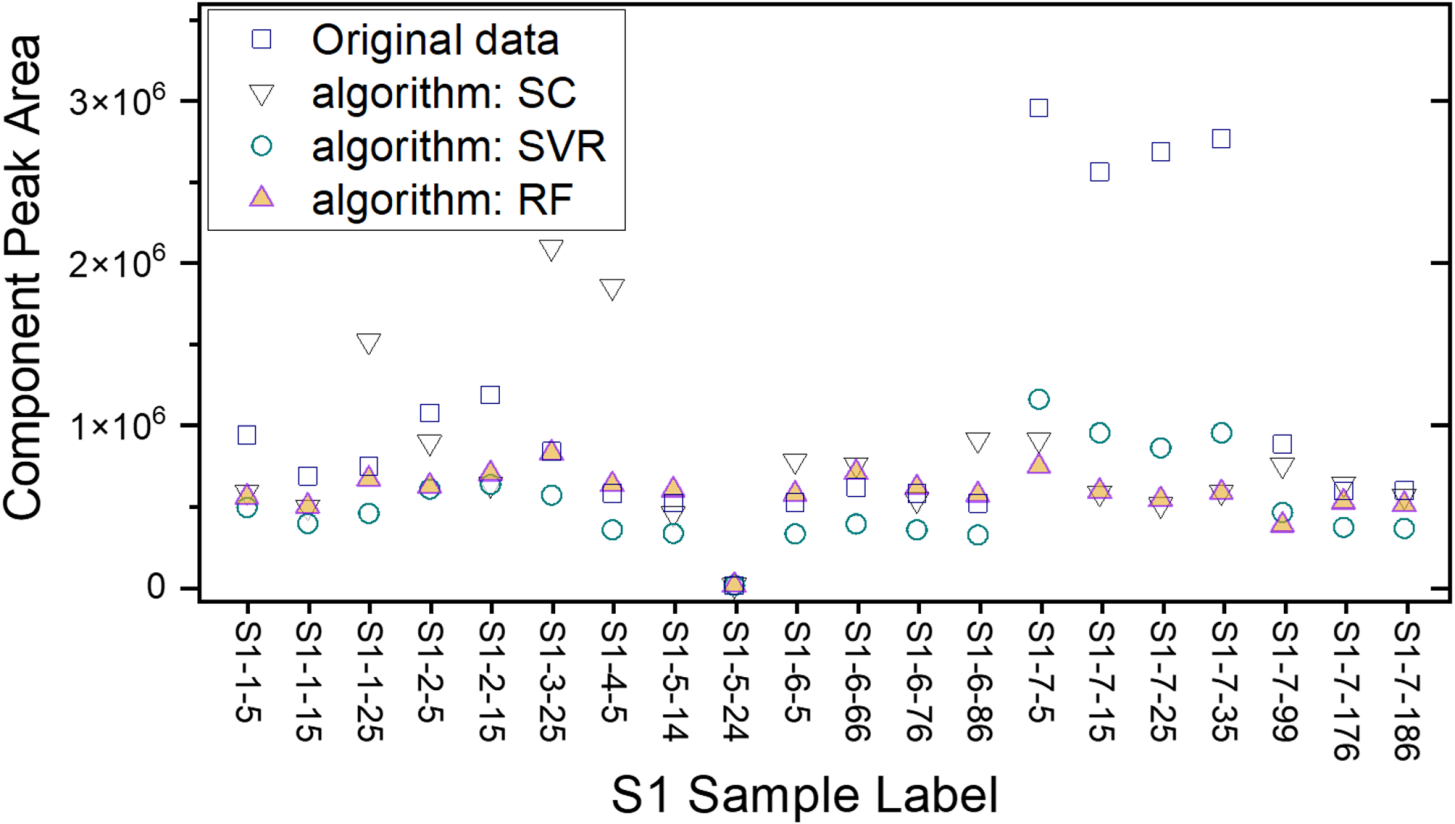
Performance of Spline Interpolation (SC), Support Vector Regression (SVR), and Random Forest (RF) on limonene component in 20 tests on S1 sample. The square dots are the raw data, the down-pointing triangles are from SC, the round dots are from SVR, and the up-pointing triangles are from RF.

The performance of the three algorithmic models on limonene in all six samples (S1 to S6) are summarised in Table 1. Statistical values in Table 1 were based on 20 tests from each sample. It is obvious that RF was better than SVR in general. Although SC performed well on some samples, the algorithm was not robust enough and it performed poorly especially on S6 data.

**Table 1:** Decrease of the standard deviation (%) of limonene component after applying Spline Interpolation (SC), Support Vector Regression (SVR), and Random Forest (RF) based correction on S1 to S6 samples.

Algorithm	S1	S2	S3	S4	S5	S6
SC	75	63	72	60	56	36
SVR	69	50	48	48	48	48
RF	81	55	79	81	77	83

We now check the performance of correction functions on all components. The components in mixed QC are not guaranteed to be detected in each sample measurement, which is due to dilution effect in pooled QC or component instability. In order to better visualize the performance of correction on different types of molecules, all sample components were separated into two groups. The first group is the components that are present in both QC and S1-S6 samples, consisting of 142 components (Group A). The second group are components existed in all samples but did not appear in the QC data, containing 36 components (Group B). Thus, Group A contained components in Category 1 mentioned before, and Group B included all components in Categories 2 and 3.

Figure 5 shows the mean of standard deviation decrease for components of Group A and Group B, for samples S1 to S6. From this figure, we conclude that all three models decreased standard deviation in both A and B groups. Correction was more effective for Group A because the decrease was larger. It is seen that SC and RF models worked better than SVR in most samples. For the SC algorithm, there are a few cases in which correction performance was comparable to RF, such as S1 and S2 in Group A. However, in general SC was slightly worse than RF. It should also be noted that SC is not very robust, especially for S6 sample. In general RF performed consistently well.

**Fig. 5:**
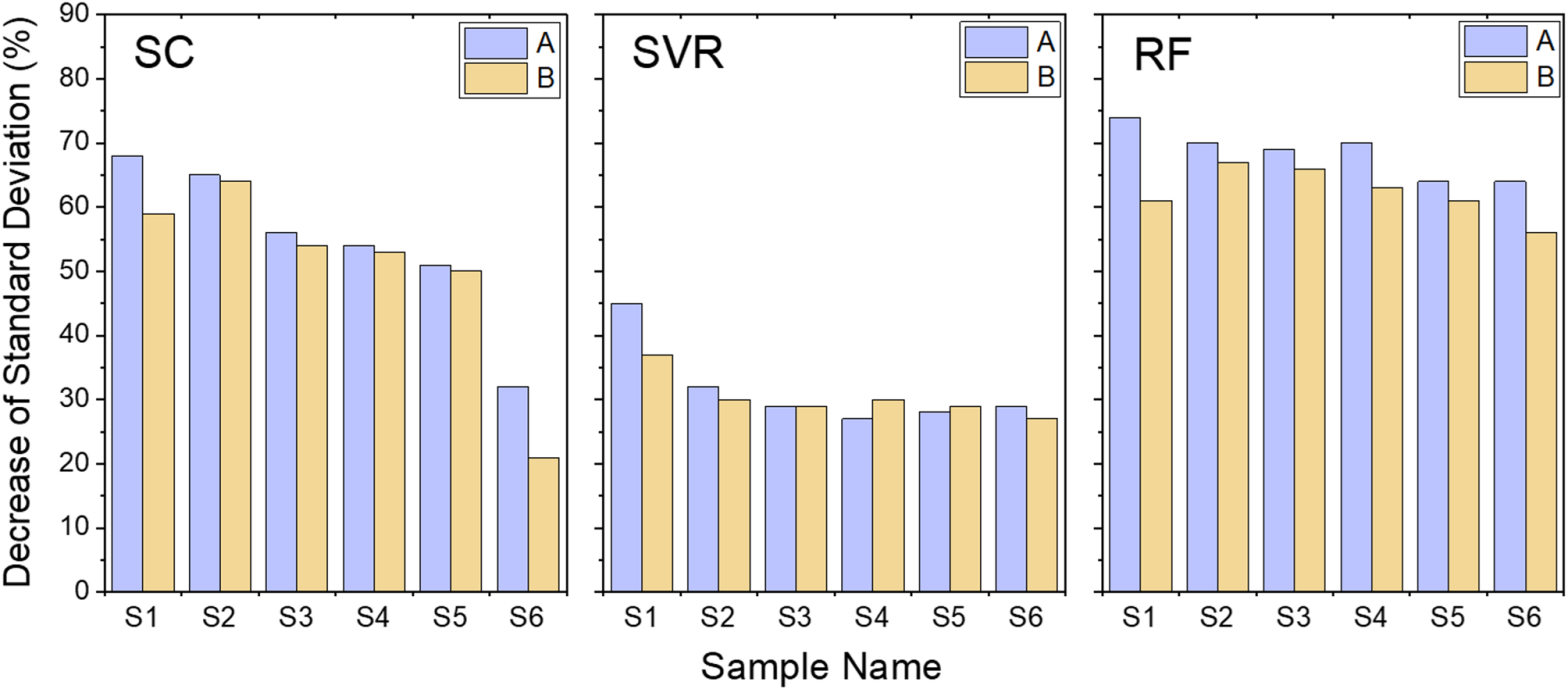
Performance of Spline Interpolation (SC), Support Vector Regression (SVR), and Random Forest (RF) on Group A components (present in QC and samples, 142 species) and on Group B components (present only in samples, not in QC, 36 species). The y-axis corresponds to the mean value of decrease of standard deviations for each sample.

In order to further explore the differences in three algorithm’s correction performance, we compared the correction coefficients of limonene on QC and samples with relatively large fluctuations. The result is shown in Fig. 6. The data were from measurements collected on days 127, 130, 131, and 134, corresponding to number 14 to 17 sets of tests. The data in Fig. 6 showed a strange pattern in the SVR-based model. It is obvious that SVR performed well for QC and reasonable for S1, but the values subsequently converged to 0.67 rapidly, which was much larger than that of QC. This is strange because for a typical component such as limonene, the correction coefficients for samples should be similar to that of QC. Hence, we concluded that SVR algorithm is prone to over-fitting for sample data with large fluctuations. The data in Fig. 6 also shows that the SC algorithm was not robust enough. Sometimes the correction coefficients were close to that of QC, but sometimes they deviated severely far away. In contrast, correction based on RF algorithm was stable for all these 28 corrections. This means correction based on RF algorithm is able to adapt to large fluctuations in a dataset.

**Fig. 6:**
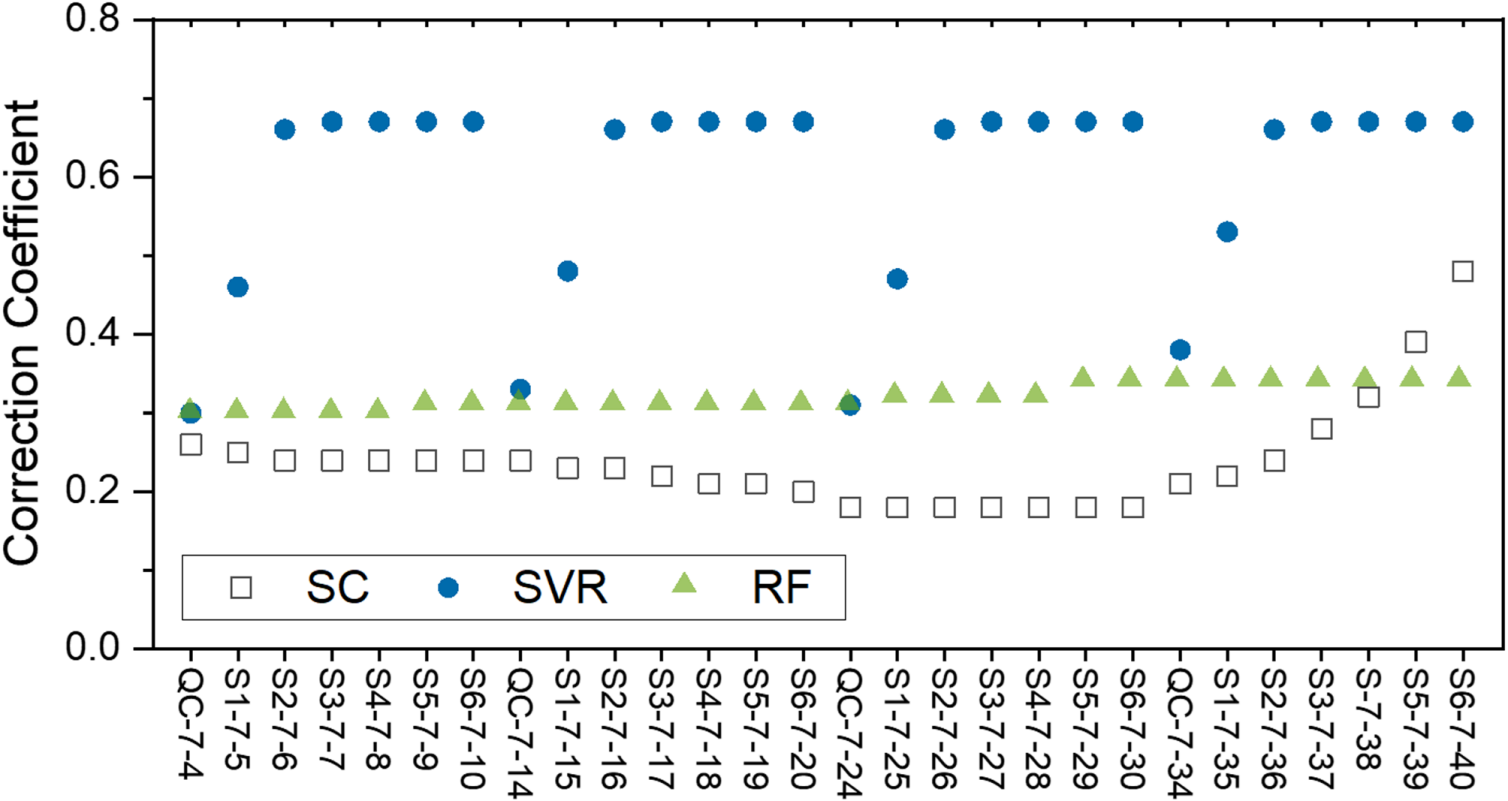
Comparison of the performance of Spline Interpolation (SC), Support Vector Regression (SVR), and Random Forest (RF) models on limonene correction coefficients in QC and samples obtained between day 127 to day 134.

### Analysis by principal component analysis

In the following, we used Principal Component Analysis (PCA) to look at the 20 measurements of sample S1 to S6 before and after correction using RF algorithm. In Fig. 7, the PCA analysis was based on all 178 components found in the sample, including 142 in Category 1 and 36 in Category 2 and 3. As can be seen in Fig. 7, data correction resulted in significant decrease of data points dispersion. The mean Euclidean distance calculated for data before correction is from 15.6 to 16.9. After correction by RF method, mean Euclidean distances dropped to 4.6–6.1.

**Fig. 7:**
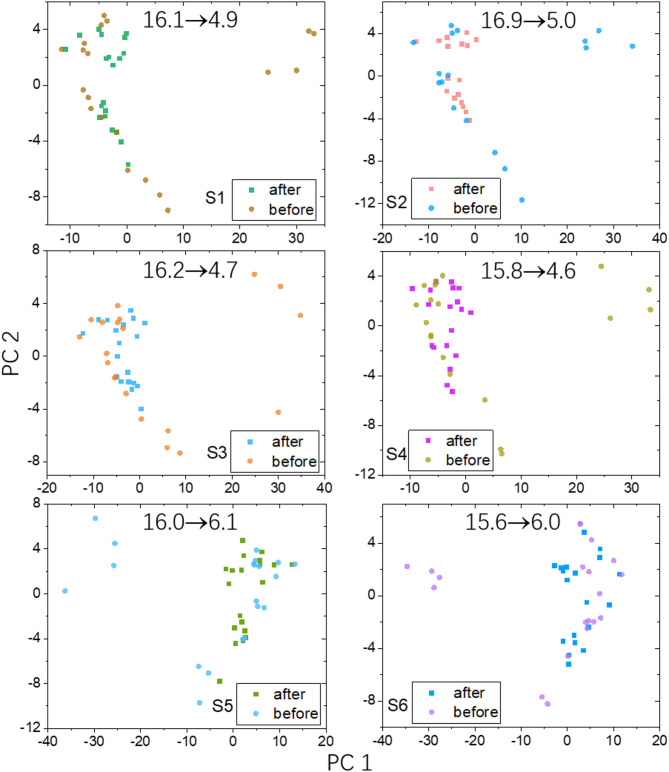
Principal Component Analysis on the performance of Random Forest (RF) correction on all the tested samples from S1 to S6. The PCA analysis used all 178 components found in samples. The mean Euclidean distances before and after data correction are given as the numbers in each plot.

### Batch effects

In Fig. 8, we use three chemical components with retention time centered at 21.01 min, 23.23 min, and 24.73 min to demonstrate two observations. First, intra-batch data fluctuation could be larger than inter-batch data fluctuation. For example, batch 2 contains 12 sample tests and has an average value of 1.36 × 10^6^ and a standard deviation of 2.3 × 10^5^. On the other hand, batches 4, 5, and 6 together give an average value of 7.7 × 10^5^ and a standard deviation of only 1.1 × 10^5^.

**Fig. 8:**
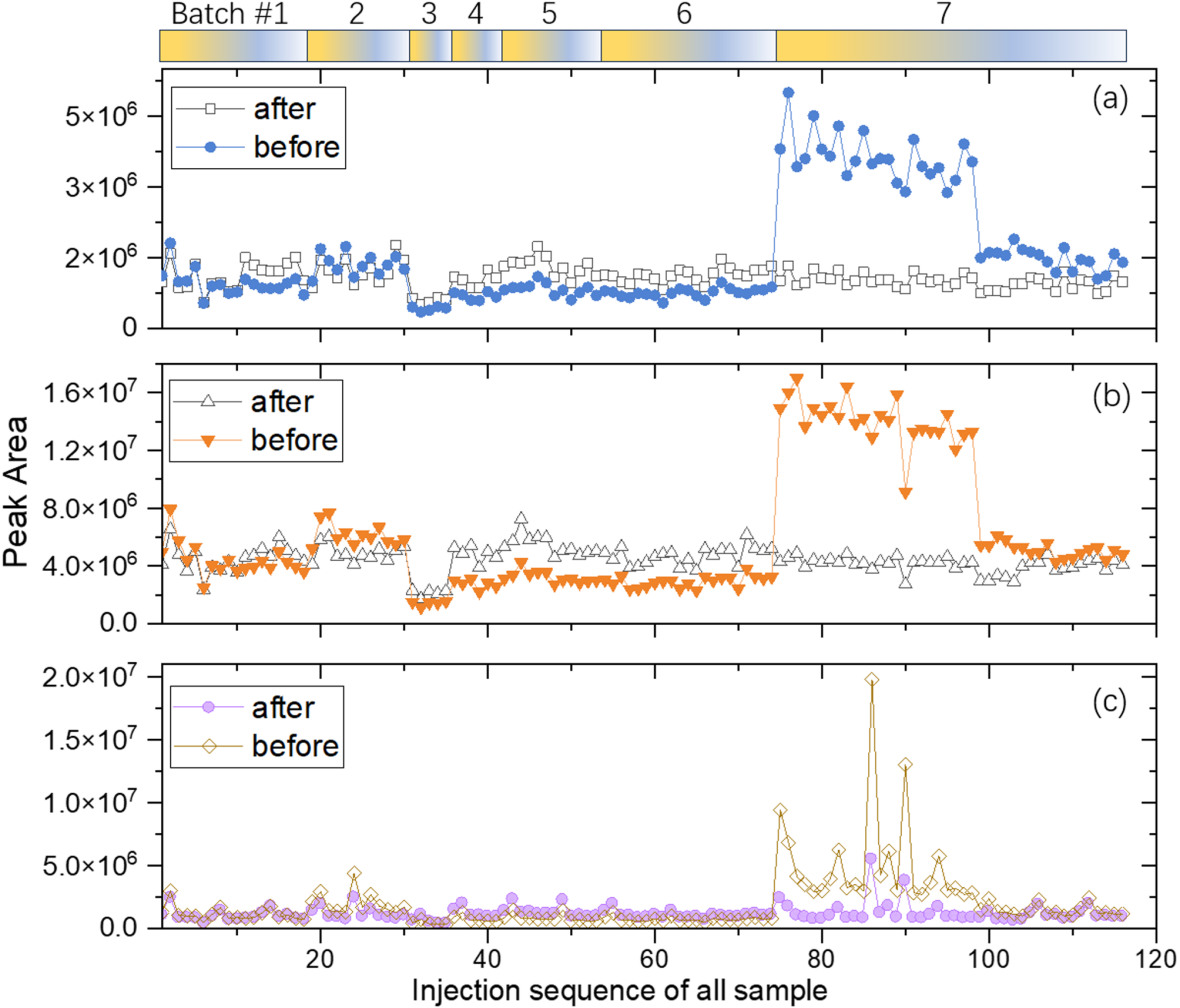
GC peak area of three chemical components with retention time at **a** 21.01 min, **b** 23.23 min, and **c** 24.73 min before and after Random Forrest correction. Data from altogether 116 samples tested within 155 days are presented sequentially. Batch numbers are giving at the top of the plot using color blocks for visualisation. Four tests (S2-6–67, S2-6–77, S2-6–87, and S4-3–28) with unreliable results are not included in this plot.

Second, inter-batch data difference can sometimes become very large as seen from batch 6 and 7. Batch 6 includes 21 tests, and the peak area (at 21.01 min) average is 7.6 × 10^5^. Batch 7 includes 42 tests, and the peak area (at 21.01 min) average is 2.7 × 10^6^.

For the component at 21.01 min, the relative standard deviations are 28% (batch 1), 17% (batch 2), 12% (batch 3), 12% (batch 4), 16% (batch 5), 13% (batch 6), and 43% (batch 7). For the component at 23.23 min, the relative standard deviations are 26% (batch 1), 12% (batch 2), 12% (batch 3), 12% (batch 4), 13% (batch 5), 13% (batch 6), 46% (batch 7). Therefore, we can see that within each batch, variation among all the sample sometimes is very large, but in the case of batch #2,3,4,5,6, the signal variation is small. This statement is true only for components with reasonably large peak amplitude, such as two at 21.01 min and 23.23 min. For the weak peak appeared at 24.73 min, peak amplitude can vary much more significantly. For example, batch #2 and 7 exhibited large intra-batch variation.

## Conclusions

In this paper, the smoke of six types of commercial cigarette samples and a pooled QC were measured 20 times by gas chromatography-mass spectrometry (GS-MS) spanning 155 days. Measured values fluctuated greatly since the instrument was not in continuous operation during this period, which experienced turn-off and turn-on, mass spectrometer tuning, ion-source cleaning, filament replacement, and quadrupole cleaning. We tested Spline Interpolation (SC), Support Vector Regression (SVR), and Random Forest (RF) algorithms, with the goal of finding optimal correction function for data in long time span. Twenty tests on pooled QC were used to establish the needed mathematical correction functions. Our findings are summarised below.

First, we proposed to construct a virtual QC for rigorous components analysis. It began with the median QC data as the starting point, and other QC results were compared with this data. All new spectral peaks were verified and added this virtual QC, which in theory contained all possible components for accurate peak alignment. Second, the data over a span of 155 days illustrated that QC indeed ensured good correction, even for fluctuating sample data over a long time. For chemical components that only present in the samples but not in the QC, these molecules could still be effectively corrected. Correction model based on Random Forest algorithm gave the best performance. In summary, we have shown that regular QC measurements with reasonable algorithm correction can effectively tackle the data fluctuation problem for long-term analysis requirement.

## Supplementary Information

Below is the link to the electronic supplementary material.

Supplementary Material 1

## Data Availability

Data is provided within the manuscript or supplementary information files. The original GC–MS data files in mzXML format are available in the public domain for research purpose. Link: 10.6084/m9.figshare.30284446.
