# Supplementary material for: Correction of gas chromatography–mass spectrometry long-term instrumental drift using quality control samples over 155 days
Source: Sci Rep. 2025 Nov 19;15:40879. doi: 10.1038/s41598-025-24794-y (PMC12630760; doi:10.1038/s41598-025-24794-y)
Supplement: Supplementary file 1 — Supplementary Material 1 [file 41598_2025_24794_MOESM1_ESM.docx]

Supporting Information

Correction of Gas Chromatography-Mass Spectrometry Long-term Instrumental Drift using Quality Control Samples over 155 days

Jie YU^1^, Tong AN^1^, Daifeng CHEN^3^, Shining ZONG^2^, Dongxiao BAI^2^, Dawei QI^1^, Luning ZHANG^2,*^& Junming SHI^3,*^

1. Technical Center, Shanghai Tobacco Group Co. Ltd., Shanghai 201315, China. 2. School of Chemical Science and Engineering, Tongji University, Shanghai 200082, China. 3. Shanghai Cigarette Factory, Shanghai Tobacco Group Co. Ltd., Shanghai 201315, China.

* Correspondence and requests for materials should be addressed to L.Z. (luningzhang@tongji.edu.cn) and J. S. (shijm@sh.tobacco.com.cn)

In Figure S1, we show the chromatograms of Quality Control samples numbered QC-1-4 and QC-7-4 which are the 1^st^ and 14^th^ QC among the 20 measurements. Notice that due to batch effect, instrument maintenance, and random error, the two results are different not only in signal magnitude, but also in the number of peaks, and retention time. Recognition of chemical species should be followed by alignment of retention time of each species, before applying any mathematical models for data correction.

Figure S1. Gas chromatography results of QC samples (a) QC-1-4 and (b) QC-7-4. The signal magnitude is calculated from each peak’s mass spectrometry ion number density.

In Figure S2, we show the GC chromatograms of 20 measurements on Quality Control samples in 33-50 min retention time. The demonstrated limonene data in Figure 3 in the main text is from this region, corresponding to peaks labeled as “*”. Some other chemicals in the eluates are also labeled with chemical structures shown. It is obvious from this figure that retention time and magnitude of the limonene peaks changed during the 155 days.

Figure S2. Gas chromatography results of 20 QC samples. The chromatography signal magnitude is from each peak’s mass spectrometry ion number density. In plot (a) from bottom to top, QC-1-4, QC-1-14, QC-1-24, QC-2-4, QC-2-14, QC-3-24, QC-4-4, QC-5-13, QC-5-23, and QC-6-4. In plot (b) from bottom to top, QC-6-65, QC-6-75, QC-6-85, QC-7-4, QC-7-14, QC-7-24, QC-7-34, QC-7-98, QC-7-175, and QC-7-185. In plot (c) chemical structures of six different species are shown, limonene is labeled as “*”. Chemical structures were obtained from mass spectrometry results using best peak fit.

In Figure S3, we show the chromatograms of 20 measurements of S1 samples in the range of 33-50 min retention time. The demonstrated limonene data in Figure 4 in the main text is from this region, with the corresponding peak labeled as “*” here. Some other chemicals in the eluates are similar to the ones shown in Figure S2.

Figure S3. Gas chromatography results of 20 measurements on S1 samples with limonene labeled as “*”. The chromatography signal magnitude is from each peak’s mass spectrometry ion number density. In plot (a) from bottom to top, S1-1-5, S1-1-15, S1-1-25, S1-2-5, S1-2-15, S1-3-25, S1-4-5, S1-5-14, S1-5-24, and S1-6-5. In plot (b) from bottom to top, S1-6-66, S1-6-76, S1-6-86, S1-7-5, S1-7-15, S1-7-25, S1-7-35, S1-7-99, S1-7-176, and S1-7-186.

In Figure S4, we show the enlarged GC results near 36 min retention time. It can be seen in this figure that signal strength varies greatly within 155 days.


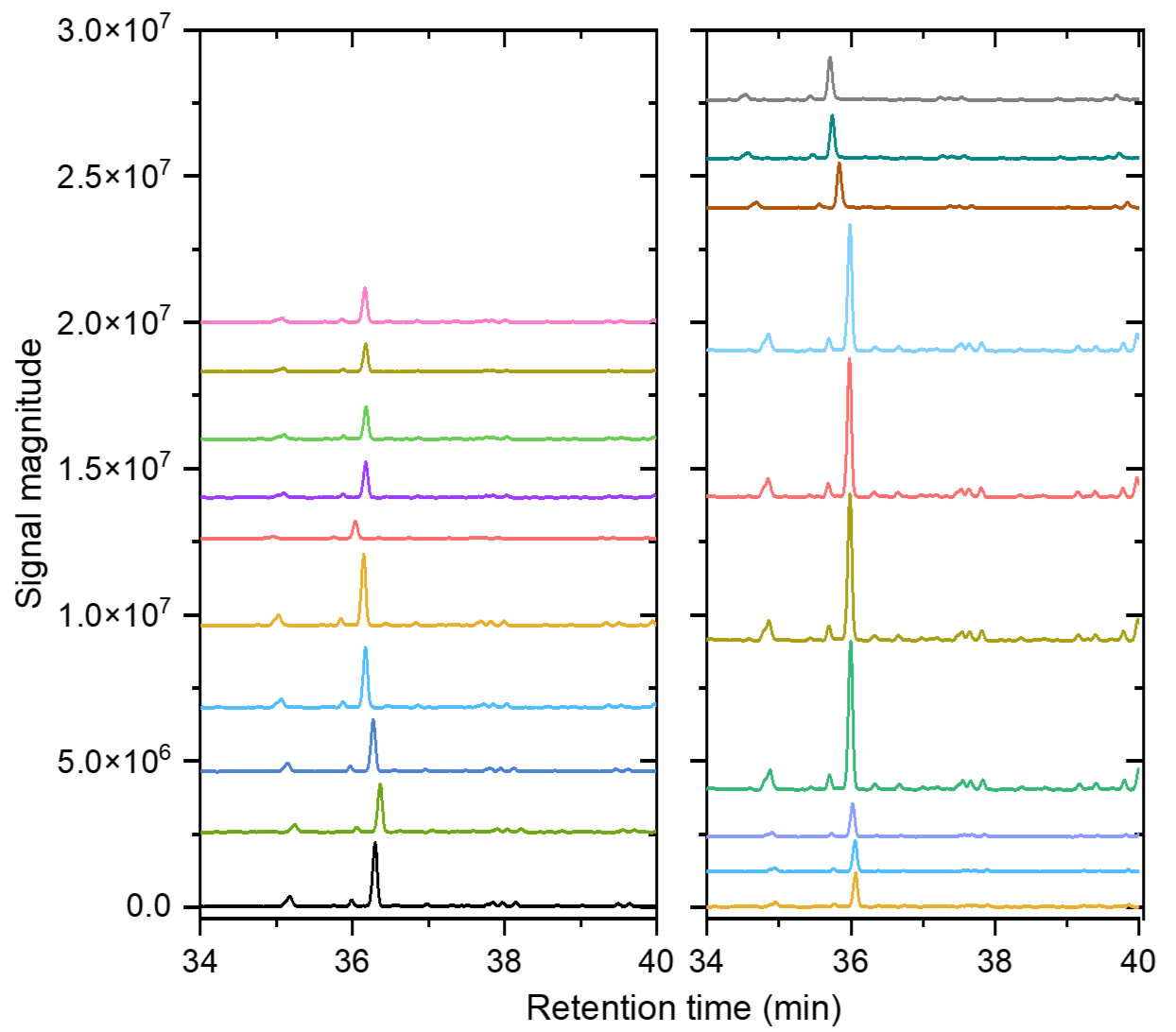


Figure S4. Gas chromatography results of 20 measurements on S1 samples with limonene at retention time around 36.1 min. The chromatography signal magnitude is from each peak’s mass spectrometry ion number density. Left panel from bottom to top, S1-1-5, S1-1-15, S1-1-25, S1-2-5, S1-2-15, S1-3-25, S1-4-5, S1-5-14, S1-5-24, and S1-6-5. Right panel from bottom to top, S1-6-66, S1-6-76, S1-6-86, S1-7-5, S1-7-15, S1-7-25, S1-7-35, S1-7-99, S1-7-176, and S1-7-186.

In Table S1, we list the corresponding labels of 20 repeated measurements on QC and S1-S6 samples, together with their testing dates in 2024 for reader’s reference.

**Table S1.** Labels of pooled QC and sample measurements. The format is a sample name followed by two numbers, which are batch number p, and injection order number t. The first column is testing date (in days), and the second column is the sequence number of 20 repeated tests. All tests were done in 2024, with the 20 repeated tests performed on the dates of (mm-dd) 03-15, 03-18, 03-20, 05-10, 05-15, 05-31, 06-05, 06-13, 06-19, 06-24, 07-08, 07-12, 07-15, 07-19, 07-22, 07-23, 07-26, 08-02, 08-14, and 08-16.

| Days | # | QC | S1 | S2 | S3 | S4 | S5 | S6 |
| --- | --- | --- | --- | --- | --- | --- | --- | --- |
| 1 | 1 | QC-1-4 | S1-1-5 | S2-1-6 | S3-1-7 | S4-1-8 | S5-1-9 | S6-1-10 |
| 2 | 2 | QC-1-14 | S1-1-15 | S2-1-16 | S3-1-17 | S4-1-18 | S5-1-19 | S6-1-20 |
| 6 | 3 | QC-1-24 | S1-1-25 | S2-1-26 | S3-1-27 | S4-1-28 | S5-1-29 | S6-1-30 |
| 57 | 4 | QC-2-4 | S1-2-5 | S2-2-6 | S3-2-7 | S4-2-8 | S5-2-9 | S6-2-10 |
| 62 | 5 | QC-2-14 | S1-2-15 | S2-2-16 | S3-2-17 | S4-2-18 | S5-2-19 | S6-2-20 |
| 78 | 6 | QC-3-24 | S1-3-25 | S2-3-26 | S3-3-27 | S4-3-28 | S5-3-29 | S6-3-30 |
| 83 | 7 | QC-4-4 | S1-4-5 | S2-4-6 | S3-4-7 | S4-4-8 | S5-4-9 | S6-4-10 |
| 91 | 8 | QC-5-13 | S1-5-14 | S2-5-15 | S3-5-16 | S4-5-17 | S5-5-18 | S6-5-19 |
| 97 | 9 | QC-5-23 | S1-5-24 | S2-5-25 | S3-5-26 | S4-5-27 | S5-5-28 | S6-5-29 |
| 102 | 10 | QC-6-4 | S1-6-5 | S2-6-6 | S3-6-7 | S4-6-8 | S5-6-9 | S6-6-10 |
| 116 | 11 | QC-6-65 | S1-6-66 | S2-6-67 | S3-6-68 | S4-6-69 | S5-6-70 | S6-6-71 |
| 120 | 12 | QC-6-75 | S1-6-76 | S2-6-77 | S3-6-78 | S4-6-79 | S5-6-80 | S6-6-81 |
| 123 | 13 | QC-6-85 | S1-6-86 | S2-6-87 | S3-6-88 | S4-6-89 | S5-6-90 | S6-6-91 |
| 127 | 14 | QC-7-4 | S1-7-5 | S2-7-6 | S3-7-7 | S4-7-8 | S5-7-9 | S6-7-10 |
| 130 | 15 | QC-7-14 | S1-7-15 | S2-7-16 | S3-7-17 | S4-7-18 | S5-7-19 | S6-7-20 |
| 131 | 16 | QC-7-24 | S1-7-25 | S2-7-26 | S3-7-27 | S4-7-28 | S5-7-29 | S6-7-30 |
| 134 | 17 | QC-7-34 | S1-7-35 | S2-7-36 | S3-7-37 | S4-7-38 | S5-7-39 | S6-7-40 |
| 141 | 18 | QC-7-98 | S1-7-99 | S2-7-100 | S3-7-101 | S4-7-102 | S5-7-103 | S6-7-104 |
| 153 | 19 | QC-7-175 | S1-7-176 | S2-7-177 | S3-7-178 | S4-7-179 | S5-7-180 | S6-7-181 |
| 155 | 20 | QC-7-185 | S1-7-186 | S2-7-187 | S3-7-188 | S4-7-189 | S5-7-190 | S6-7-191 |

In Figure S5, we show the original and corrected data for two low abundance components in S1 sample. The two peaks correspond to toluene and pyridine with retention time of 24.73 min and 34.94 min, respectively. As is shown in Figure S5(a), limonene peak (with *) is much larger than these two components. In Figure S5(b) and (c), we can see that RF and SVR algorithms perform well. The standard deviation of toluene before correction is 2.1×10^6^, and after correction it has become 3.2×10^6^ (SC), 8×10^5^ (SVR), and 4×10^5^ (RF). The standard deviation of pyridine before correction is 3.5×10^6^, and after correction it has become 2.3×10^6^ (SC), 1.3×10^6^ (SVR), and 9×10^5^ (RF).


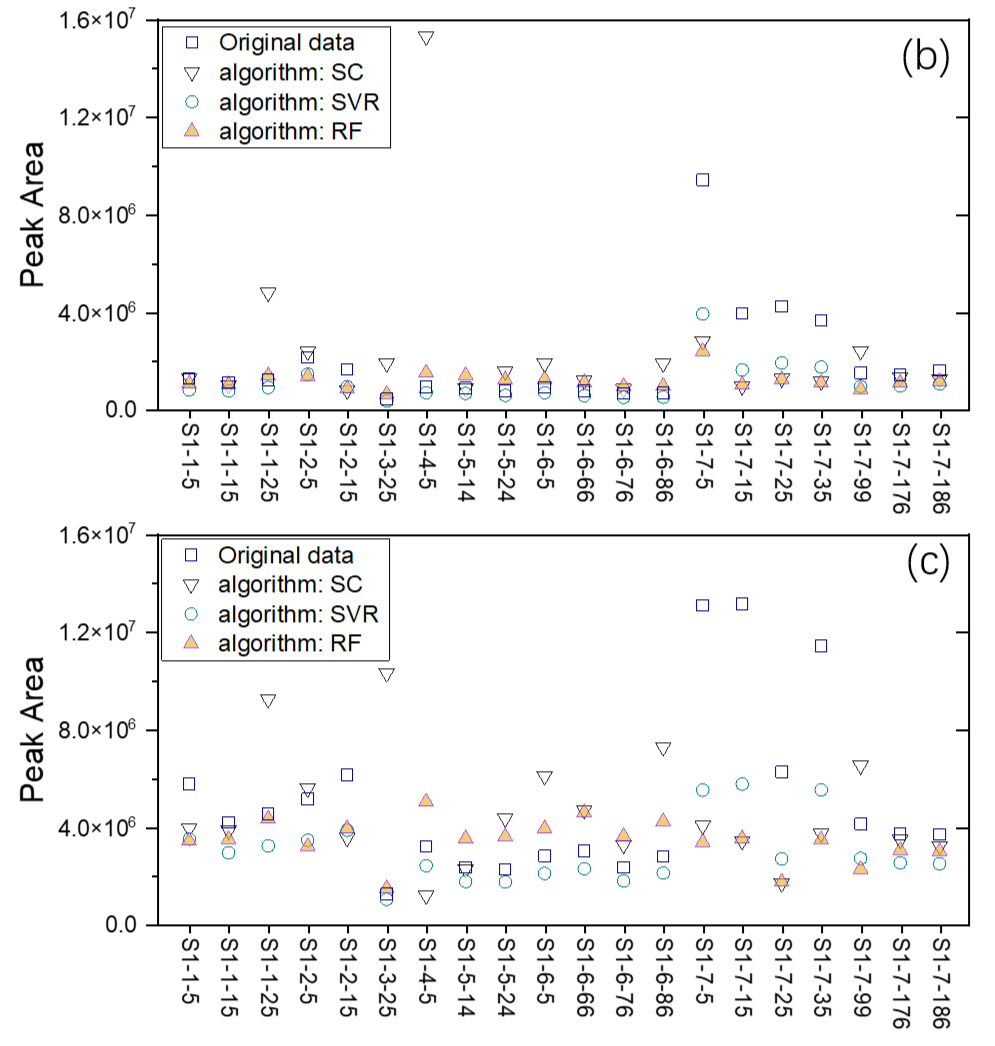

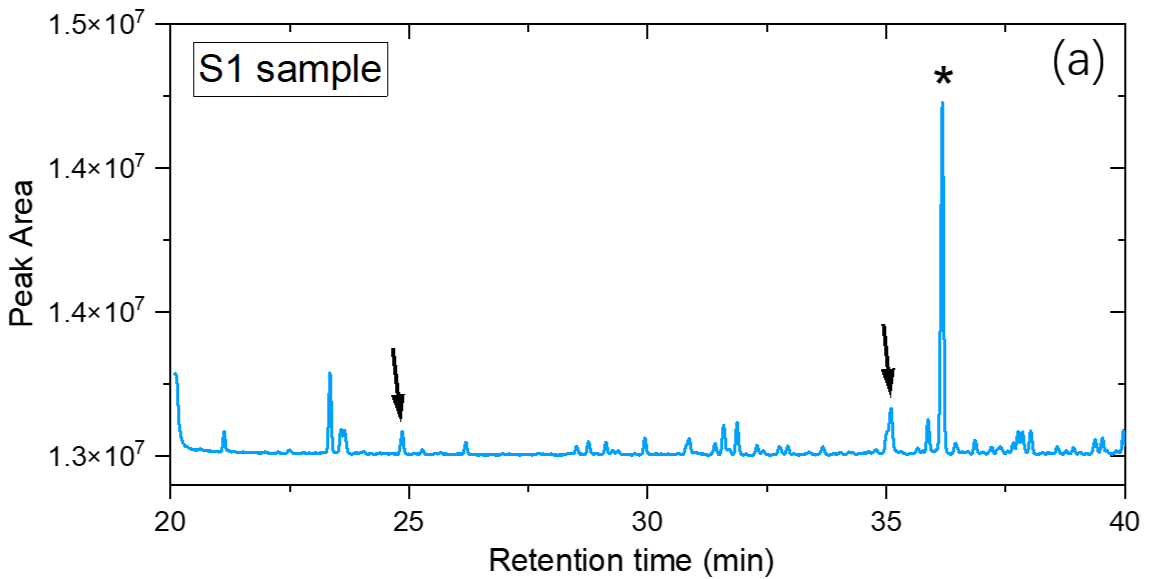


Figure S5. Performance of Spline Interpolation (SC), Support Vector Regression (SVR), and Random Forest (RF) on two low abundance components of Category 1 of S1. (a) GC result with arrows indicating two analysed peaks, and limonene labeled with “*”. Peak at 24.73 min is toluene, and peak at 34.94 min is pyridine. (b) Original and corrected data for toluene. (c) Original and corrected data for pyridine.

From the above analysis, we hope to show that for low abundance components in Category 1, with peak intensity about 5%~10% of limonene peak, the three algorithms performed differently and the difference among them increased. For example, the percentage decrease of standard deviation for limonene after correction was 75% (SC), 69% (SVR), and 81% (RF). The percentage change of standard deviation for toluene was 62% (SVR), and 81% (RF). The standard deviation erroneously increased when using SC algorithm on toluene.

While for pyridine, the change was 34% (SC), 63% (SVR), and 75% (RF). For low abundance components such as toluene and pyridine, RF is more reliable and perform best while SVR is mediocre. However, SC is not consistent and sometimes it increased standard deviation after correction. Similar analysis of other samples gave us same conclusion for low abundance components. The take-home message is that RF is reliable


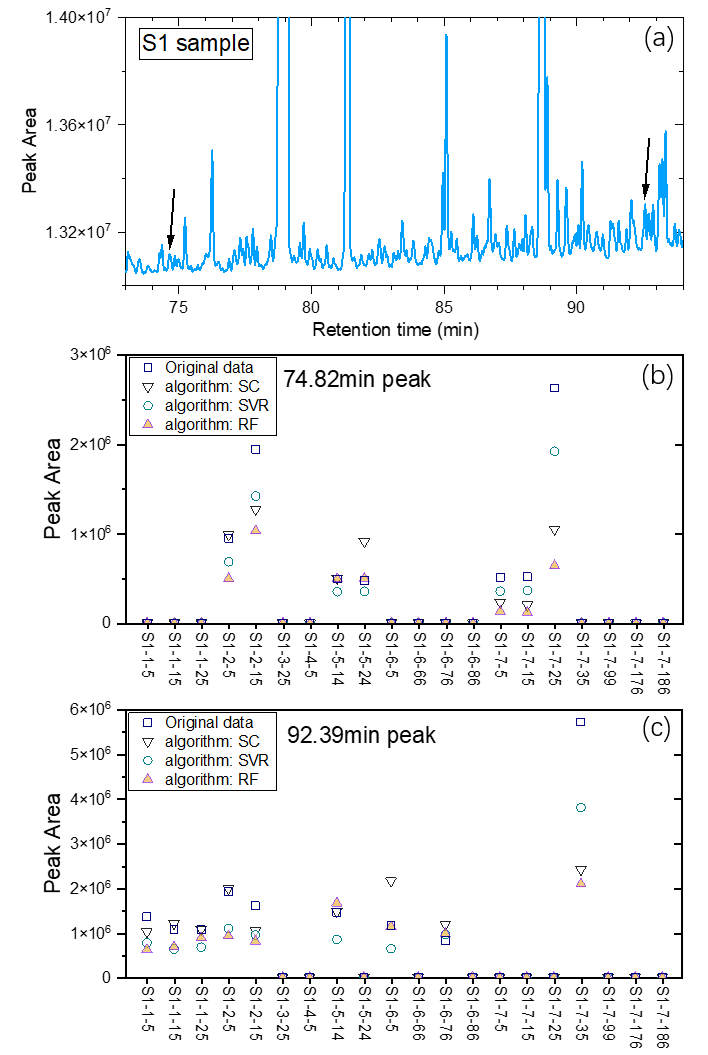


Figure S6. Performance of Spline Interpolation (SC), Support Vector Regression (SVR), and Random Forest (RF) on two low abundance components of Category 2 of S1. (a) GC result with arrows indicating two analysed peaks. (b) Original and corrected data for component at 72.82 min. (c) Original and corrected data for component at 92.39 min.

In Figure S6, we show two typical chemicals in Category 2. They show up some of the S1 samples, but do not find any mass spectrum match within QC samples. The first peak is at 74.82 min which is assigned to 1-nonanol based on its mass spectrum. It does not match the chemicals in QC. A nearby peak at 74.98min in QC was endo-Borneol which can be used for 1-nonanol correction for S1 samples. The second peak is at 92.39 min which is assigned to 3-pyridinol in S1. It does not match the chemical at the same location in the QC sample. In QC, the peak at 92.36 min is p-Cymene. So when doing correction for 3-pyridinol in S1, we need to take the corresponding correction function and coefficient for p-Cymene in QC.

In Figure S7, we show the correction results for a typical Category 3 component (2-methyl-3-phenylpropanal), using S1 samples for demonstration. This molecule does not show up in the QC data, but shows up in some S1 and S4 samples. In S1 samples, we can see that among the 20 tests, 8 of them exhibit this peak. Analysis on the 8 tests reveals that the original peak area is 1.10×10^6^ (SD=0.69×10^6^). After correction, we get the following peak area and SD: 1.25×10^6^ (SD=0.58×10^6^) for RF, 0.86×10^6^ (0.52×10^6^) for SVR, and 2.48×10^6^ (SD=1.86×10^6^) for SC. We can conclude that SC perform badly in this case since the SD became unreasonably high. Both RF and SVR work well with RF better than SVR.


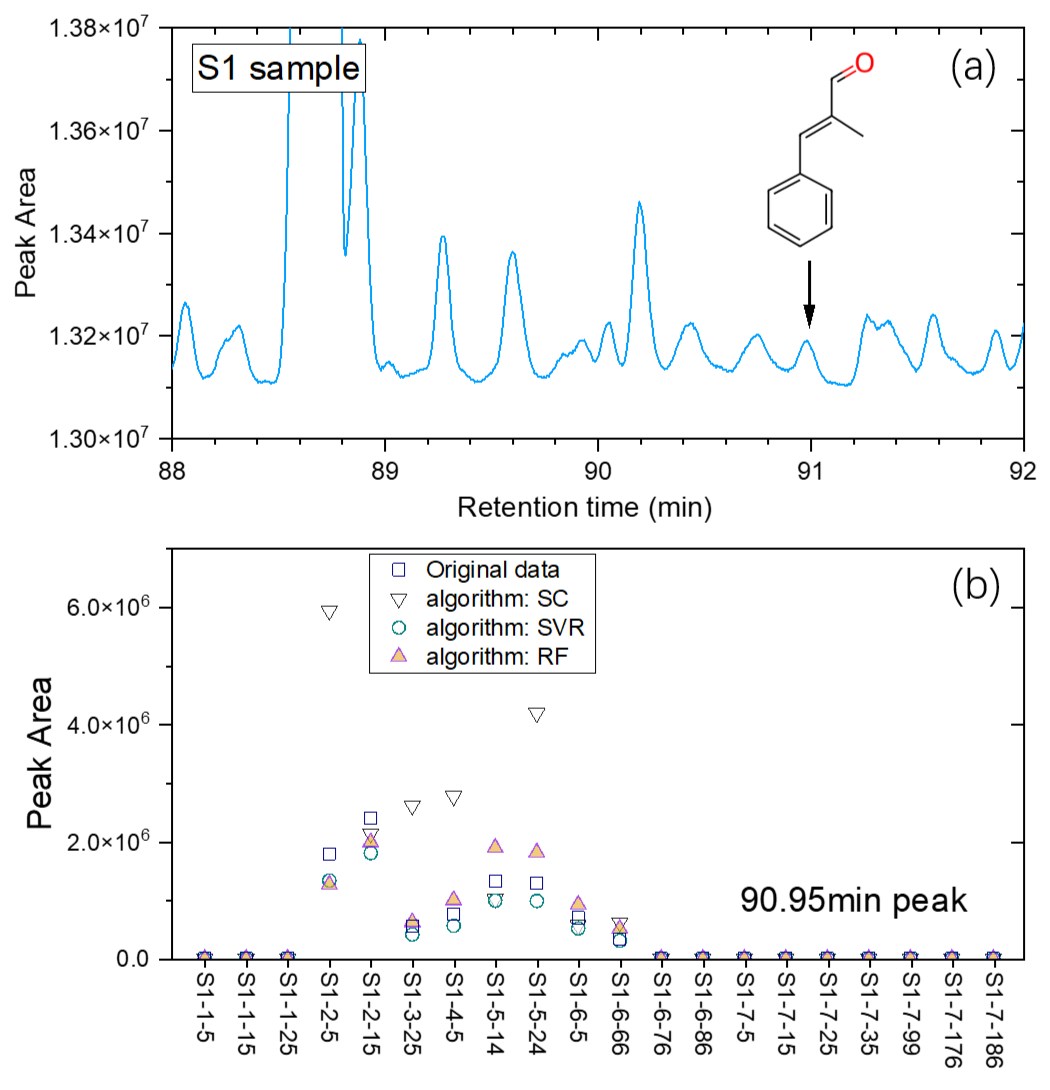


Figure S7. Performance of Spline Interpolation (SC), Support Vector Regression (SVR), and Random Forest (RF) on a low abundance component of Category 3 of S1. (a) GC result with arrow indicating analysed peak position. (b) Original and corrected data for component at 90.95 min.


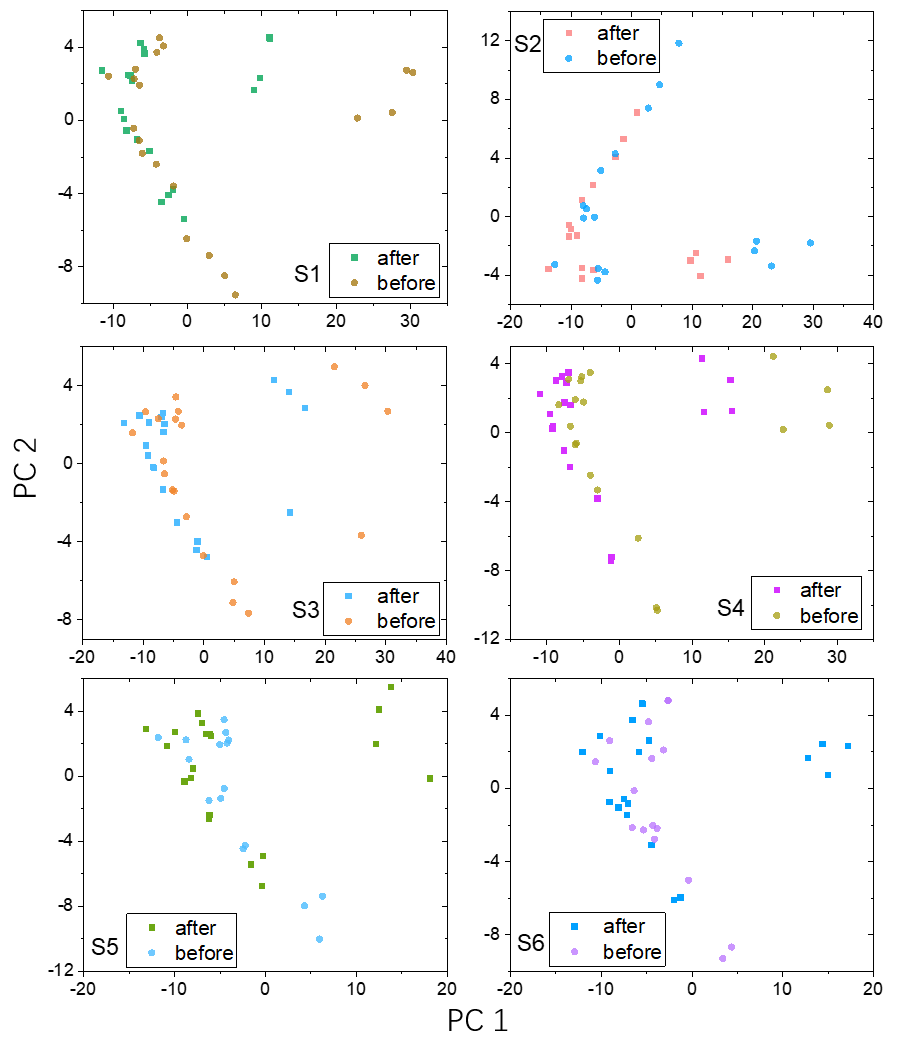


Figure S8. Principal Component Analysis on the performance of SVR correction on all the tested samples from S1 to S6. The PCA analysis used all 178 components found in samples.
